# Effect of Vaping on Treatment Outcomes in Patients With Gingivitis, Periodontitis, and/or Peri‐Implant Disease: A Systematic Review

**DOI:** 10.1002/cre2.70456

**Published:** 2026-09-15

**Authors:** Stacey May Gould, Catherine Anne Gilchrist, Hannah Dominique Gaza, Ibadat Kaur Sidhu, Kayleigh Jade Hugo, Rodrigo Rodrigues Amaral, João Martins de Mello‐Neto

**Affiliations:** ^1^ College of Medicine and Dentistry James Cook University, Campus Smithfield Cairns Queensland Australia; ^2^ Australian Institute of Tropical Health and Medicine James Cook University Townsville Australia; ^3^ Department of Endodontics Nova Southeastern University, College of Dental Medicine Fort Lauderdale Florida USA

**Keywords:** E‐cigarette, peri‐implant disease, periodontal disease, vape

## Abstract

**Objective:**

This systematic review aimed to evaluate the effect of vaping on periodontal treatment outcomes for patients with gingivitis, periodontitis, and/or peri‐implant disease.

**Material and Methods:**

An electronic database search was conducted using Ovid MEDLINE, PubMed, Scopus, and Web of Science, with no date restrictions. An electronic search was conducted through June 30, 2026. Two independent reviewers extracted data according to predefined inclusion criteria and performed screening and quality appraisal. The risk of bias was assessed using the Newcastle‐Ottawa Quality Assessment Scale and the Cochrane Risk of Bias tool.

**Results:**

Seven studies were included, involving 640 participants: 168 vapers, 138 traditional cigarette smokers, and 334 non‐smokers. There was no significant difference in clinical response to periodontal and peri‐implant therapy between tobacco and e‐cigarette users, both of whom fared worse than non‐smokers. Immunological outcomes were similar between smokers and vapers, with some exceptions, such as higher TNF‐α levels in e‐cigarette users. No meta‐analysis was performed due to the heterogeneity of the included studies.

**Conclusion:**

Current limited evidence suggests that vaping may be associated with less favorable periodontal and peri‐implant treatment outcomes compared with non‐smoking, with trends resembling those observed in cigarette smokers. Further research is required to clarify the extent to which vaping affects treatment outcomes compared to cigarette smokers.

## 1 Introduction

Electronic cigarettes (e‐cigarettes or vapes) are battery‐operated devices that vaporize a solution of nicotine, flavoring agents, humectants, and additives like propylene glycol and vegetable glycerine (Javed, Kellesarian, et al. 2017; Kaisar et al. 2016). Vaping has rapidly gained popularity, especially among adults aged 18–24, with 11%–21% of smokers in the United States reporting e‐cigarette use (Javed, Kellesarian, et al. 2017). In Italy, the prevalence of exclusive vaping among 13–15‐year‐olds tripled from 2.9% to 8.2% between 2014 and 2018 (Gorini et al. 2020). Usage is also increasing in Southeast Asia, particularly among adolescents (Serra et al. 2023).

The rapid adoption of vapes is often fuelled by the belief that they are a safer alternative to traditional cigarettes and can aid smoking cessation, despite conflicting evidence (Kaisar et al. 2016; Adam and Hasan 2023). However, concerns persist regarding the lack of knowledge about the toxicological effects of vaping and its potential to act as a gateway to smoking in younger populations (Kaisar et al. 2016; Adam and Hasan 2023).

Cigarette smoking is well‐known to increase the risk of systemic conditions and negatively impact oral health, including periodontitis, peri‐implantitis, and oral cancer (Ramôa et al. 2017). Smoking also impairs the response to periodontal therapy (Ramôa et al. 2017). While the adverse effects of vaping on health are less well understood, its long‐term safety remains unquantified (Sun et al. 2022). As vape usage rises globally, dental professionals are likely to encounter more vape users, highlighting the need for further research on the long‐term effects of vaping on oral and systemic health (Abbott et al. 2023).

Periodontal diseases, encompassing gingivitis, periodontitis, and peri‐implant diseases (peri‐implant mucositis and peri‐implantitis), present a significant worldwide public health concern (Nocini et al. 2020). These conditions, characterized by inflammation and destruction of supporting tooth or implant structures, affect oral health and have broader systemic implications (Nocini et al. 2020; Caton et al. 2018). Gingivitis is the mildest form of periodontal disease, which affects the gingival tissues (Nocini et al. 2020; Caton et al. 2018; Gingivitis 2024). In contrast, periodontitis results in periodontal pocketing, clinical attachment loss, and alveolar bone loss, and may lead to tooth loss (Caton et al. 2018; Periodontitis 2024; Tonetti et al. 2018). Similar to gingivitis, peri‐implant mucositis is characterized by inflammation confined to the soft tissues surrounding a dental implant with no loss of supporting bone; in contrast, peri‐implantitis is an inflammatory process that involves both inflammation of the mucosa and loss of supporting bone (Caton et al. 2018; Gingivitis 2024; American Academy of Periodontology 2024, 2012).

Periodontal therapy focuses on preserving natural teeth, periodontium, and peri‐implant tissues through techniques that reduce inflammation and promote attachment (Nevins 2025). Treatments range from non‐surgical root instrumentation to surgical options like flap debridement and tissue regeneration (Kwon et al. 2021; Caffesse and Echeverría 2019). Adjunctive therapies, including antimicrobials and probiotics, can also enhance outcomes (Kwon et al. 2021; Caffesse and Echeverría 2019; RASHMİ DURGA et al. 2023).

The impact of cigarette smoking on the onset and progression of periodontal disease is well‐documented, primarily through its effects on the host immune response and the release of pro‐inflammatory cytokines (Charde et al. 2024). In contrast, the impact of vapes on oral cytokine levels remains less well studied (Faridoun et al. 2021). Existing research indicates that cigarette smokers exhibit higher levels of prostaglandin E2 (PGE2) and interleukin 1‐beta (IL‐1β) in periodontal disease patients, compared to vape users (Faridoun et al. 2021). More recently, it has been shown that vape use has acute negative effects, causing endothelial dysfunction and vascular and cerebral oxidative stress (Sun et al. 2022). E‐vapor, when in direct contact with oral cells, can damage the periodontal ligament and the gingival fibroblast (Javed, Kellesarian, et al. 2017). The detrimental effects are due to the generation of reactive oxygen species from e‐vapor, leading to DNA damage (Javed, Kellesarian, et al. 2017). A systematic review by Pesce et al. presented evidence suggesting that vaping is associated with increased periodontal inflammation and compromised treatment outcomes (Pesce et al. 2022). Furthermore, D'Ambrosio et al. reported higher rates of post‐treatment complications, such as delayed healing and increased recurrence of periodontal disease among vape users compared to non‐users (D'Ambrosio et al. 2022).

With the growing prevalence of vaping, understanding its impact on periodontal and peri‐implant treatment outcomes is essential (Thiem et al. 2023). While cigarette smoking is a well‐established risk factor for these conditions, the specific effects of vaping remain unclear (Caffesse and Echeverría 2019; Thiem et al. 2023; Madi et al. 2023; Naji et al. 2020). This systematic review aims to comprehensively evaluate existing evidence to elucidate the relationship between vaping and the efficacy of periodontal and peri‐implant treatment in patients with gingivitis, periodontitis, or peri‐implant disease. Ultimately, this knowledge can guide evidence‐based strategies for the management of periodontal and peri‐implant diseases in an era where vaping has become increasingly prevalent.

## 2 Materials and Methods

### 2.1 Study Protocol and Registration

This systematic review complies with the Preferred Reporting Items for Systematic Reviews and Meta‐Analyses (PRISMA) statement guidelines (Page et al. 2021). The protocol for this review was registered in PROSPERO (REF: CRD42024506111).

### 2.2 Study Objectives

This study aimed to systematically review the published literature (i.e., primary studies) regarding the effects of vaping on periodontal and peri‐implant treatment outcomes. The PICO question “In subjects with gingivitis, periodontitis, or peri‐implant disease who vape (P), does undergoing periodontal or peri‐implant treatment (I), compared to those who are cigarette smokers, former smokers, or non‐smokers (C), result in better clinical and immunological outcomes in terms of periodontal and peri‐implant parameters (O)?” was applied.

### 2.3 Search Strategy

A comprehensive search was conducted using electronic databases, including Ovid MEDLINE, PubMed, Scopus, and Web of Science. Two independent reviewers (H.D.G and S.M.G) conducted an electronic search through June 30, 2026, using Medical Subject Headings (MeSH) and other free terms or keywords. The search strategy combined keywords related to vaping, gingivitis, periodontitis, peri‐implant disease, and periodontal treatments (e.g., nonsurgical periodontal therapy [NSPT] and periodontal surgery). Appropriate Boolean operators (OR, AND) were used. The search was limited to studies published in English. No publication date limitations were applied. The identification of additional literature was completed using hand‐searching of systematic review reference lists and using artificial intelligence databases. Perplexity AI and Elicit were used as supplementary tools for literature discovery following completion of the database searches. Queries were based on the same key concepts and keywords used in the formal search strategy. References suggested by these tools were reviewed manually, and all potentially relevant studies were independently verified by examining bibliographic information, abstracts, and full texts when necessary. Studies identified through Perplexity or Elicit were subjected to the same eligibility criteria and screening procedures as those applied to records retrieved from the primary databases. The use of these tools was intended to supplement, rather than replace, conventional database searching (Perplexity, Elicit). The MeSH and keywords used across all databases are presented in Supporting Information S1: Tables S1 and S2.

### 2.4 Study Selection and Eligibility Criteria

Studies were considered for inclusion if they met the following criteria: (1) participants used vape products; (2) participants had either gingivitis, periodontitis, or peri‐implant disease; and (3) intervention involved periodontal or peri‐implant therapy (surgical or nonsurgical). Exclusion criteria included studies not published in English, non‐human studies, reviews, no use of vaping products, no mention of oral disease (gingivitis, periodontitis, peri‐implant disease), no mention of periodontal or peri‐implant therapy, studies lacking relevant outcome data, and studies where the full‐text was not retrievable. Further exclusion of Tobacco Heating Systems (THS), waterpipe use, and the lack of non‐smokers or cigarette smokers in the control data was completed. No restrictions were placed on participants' age, gender, or ethnicity.

### 2.5 Data Extraction

All identified articles were retrieved into EndNote, where duplicates were removed. The abstracts were screened by two independent reviewers (I.K.S. and K.J.H.), and the articles deemed relevant were reviewed in full. Disagreement between reviewers was resolved through discussion, and a third reviewer (J.M.M.N.) was consulted when an agreement could not be reached. Pre‐piloted forms were independently used for data extraction by two researchers (H.D.G. and S.M.G.), and the data were checked for accuracy by a third reviewer (J.M.M.N.). A standard table outlining data items and definitions was created in Microsoft Excel. Recording of data included study characteristics (author, publication year, study design), participant demographics (age, gender), intervention details (type of periodontal or peri‐implant therapy used), identification of additional groups, including control group or comparison group details, outcomes (clinical and/or immunological parameters), and follow‐up duration. The final articles were selected by the same reviewers, and any discrepancies between reviewers were resolved through discussion or consultation with an additional reviewer (J.M.M.N.).

### 2.6 Quality Assessment

Inter‐rater reliability was quantified using Cohen's Kappa Coefficient, which assesses consistency in judgments between reviewers (McHugh 2012). The quality of included studies was assessed using the Cochrane Risk of Bias (ROB‐2) tool for randomized control trials (Sterne et al. 2019) and the Newcastle‐Ottawa Quality Assessment Scale (NOS), modified for cohort, case‐control, and cross‐sectional studies (Wells et al. 2024a, 2024b). The ROB‐2 was used in one randomized controlled trial and assessed various domains of potential bias, including risk arising from the randomization process, risk of bias due to deviation from the intended intervention, missing outcome data, risk of bias in the measurement of outcomes, and risk of bias in the selection of reported results (Sterne et al. 2019). The domains assessed in the NOS included bias related to selection, comparability, exposure, analysis, reporting, and other sources specified in risk of bias manuals (Wells et al. 2024a, 2024b). For cohort and case‐control studies, up to two stars could be awarded for the comparability section only, and for cross‐sectional studies, two stars could be awarded in both selection and exposure/outcome sections only, with a maximum of nine stars awarded in total. Studies were given an overall numerical value to indicate the risk of bias for each domain and allocated terms “good,” “fair,” or “poor,” resulting in a hierarchical classification (Wells et al. 2024a, 2024b). These assessments were completed by two independent reviewers (H.D.G. and C.A.G.).

### 2.7 Data Synthesis

Data synthesis involved a descriptive/narrative synthesis of study findings due to heterogeneity in study designs and outcome measures. Subgroup analyses were planned to explore variations in the treatments completed for patients with gingivitis, periodontitis, and/or peri‐implant diseases and whether quantity and duration of vape use affected treatment outcomes. Meta‐analysis was considered; however, the results were heterogeneous.

## 3 Results

**Figure 1:**
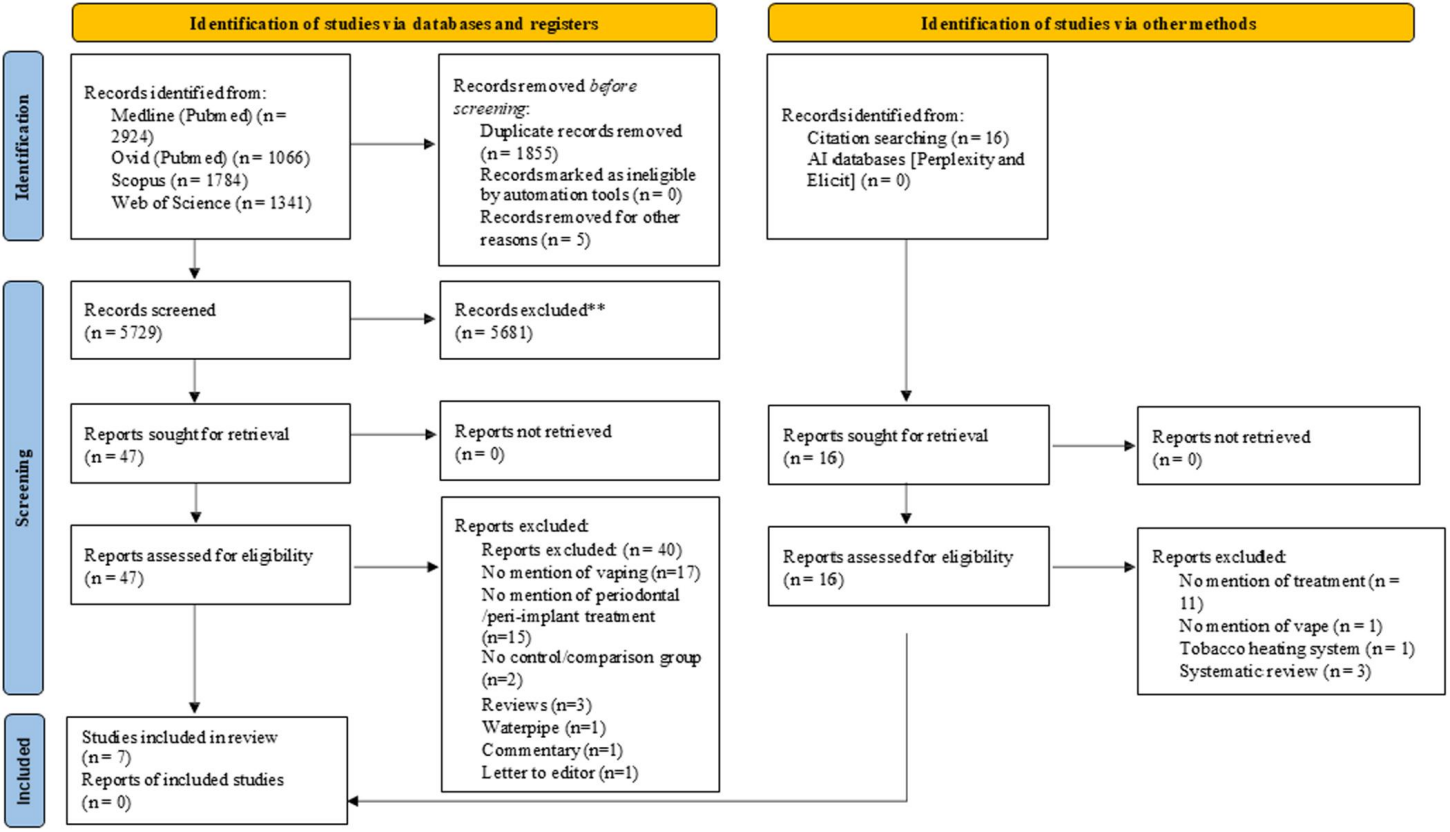
PRISMA flowchart for the electronic search of the databases.

The electronic search of the databases identified a total of 7591 records as follows: Medline/Pubmed (*N* = 2924), Medline/Ovid (*N* = 1066), Scopus (*N* = 1784), and Web of Science (*N* = 1341). A total of 1855 duplicates were identified and removed using EndNote, and the remaining 5729 abstracts were screened by two independent reviewers (I.K.S. and K.J.H.). A total of 47 full texts were reviewed, and seven articles were included in the qualitative analyses based on eligibility (Figure 1) (Al Deeb et al. 2020a, 2020b; Al‐Hamoudi et al. 2020; ALharthi et al. 2019; Alhumaidan et al. 2022; AlJasser et al. 2021; Shah et al. 2023). Inter‐rater reliability was substantial, with a Cohen's kappa coefficient of 0.80 for both screening steps, indicating almost perfect agreement beyond chance between reviewers (McHugh 2012).

### 3.1 Demographics of Participants in Included Studies

In total, four cohort studies (Al Deeb et al. 2020b; ALharthi et al. 2019; Alhumaidan et al. 2022; Shah et al. 2023), one case‐control study (AlJasser et al. 2021), one cross‐sectional study (Al‐Hamoudi et al. 2020), and one randomized control trial (Al Deeb et al. 2020a), were included in this systematic review (Table 1). A total of 640 participants were included in the reviewed studies, consisting of 450 males (Al Deeb et al. 2020a, 2020b; Al‐Hamoudi et al. 2020; ALharthi et al. 2019; Alhumaidan et al. 2022; AlJasser et al. 2021; Shah et al. 2023). Of this, 168 participants exclusively vape (more than 1‐year), (Al Deeb et al. 2020a, 2020b; Al‐Hamoudi et al. 2020; ALharthi et al. 2019; Alhumaidan et al. 2022; AlJasser et al. 2021; Shah et al. 2023) 138 participants exclusively smoke cigarettes (more than 1‐year), (Al Deeb et al. 2020a, 2020b; ALharthi et al. 2019; Alhumaidan et al. 2022; AlJasser et al. 2021; Shah et al. 2023) 334 were reported as non‐smokers (Al Deeb et al. 2020a, 2020b; Al‐Hamoudi et al. 2020; ALharthi et al. 2019; Alhumaidan et al. 2022; AlJasser et al. 2021; Shah et al. 2023). The age of vape users had a mean reported range from 27.8 to 54.1 years (Al Deeb et al. 2020a, 2020b; Al‐Hamoudi et al. 2020; ALharthi et al. 2019; Alhumaidan et al. 2022; AlJasser et al. 2021; Shah et al. 2023). The frequency of vape use in the form of “sessions per day” was recorded in five studies, with a mean frequency of 4.1–17.6 sessions (Al Deeb et al. 2020a, 2020b; Al‐Hamoudi et al. 2020; ALharthi et al. 2019; AlJasser et al. 2021). Duration of vape use was reported in six studies, with a mean range from 3.1 to 6.8 years (Al Deeb et al. 2020a, 2020b; Al‐Hamoudi et al. 2020; ALharthi et al. 2019; AlJasser et al. 2021). Comparatively, cigarette users had a mean range of 8.3–12.6 cigarettes smoked per day and a mean duration of use of 7.6–10.4 years, reported in five studies, (Al Deeb et al. 2020a, 2020b; ALharthi et al. 2019; AlJasser et al. 2021; Shah et al. 2023) and three studies, respectively (Al Deeb et al. 2020a, 2020b; ALharthi et al. 2019). Three studies provided pack‐years as a form of duration of cigarette use (Alhumaidan et al. 2022; AlJasser et al. 2021; Shah et al. 2023). Analysis of the geographic distribution revealed that five of the studies were carried out in Saudi Arabia, (Al Deeb et al. 2020a, 2020b; Al‐Hamoudi et al. 2020; Alhumaidan et al. 2022; AlJasser et al. 2021) one in the United Kingdom (Shah et al. 2023), and one was unspecified (ALharthi et al. 2019).

**Table 1:** Study information regarding year, design, assessment of health status, sample size, sex, age, disease and severity, frequency, and duration.

Author (year), country	Study design	Assessment of health status of participants	Sample size (*N*) and sex	Age (years), mean (SD)	Disease and severity			Frequency (per day), mean (SD)	Duration (years), mean (SD)
					Gingivitis (and severity)	Periodontitis (and severity)	Peri‐implant disease (and severity)		
Al Deeb et al. (2020a), Saudi Arabia	Randomized control trial	PI, BoP, PD Digital periapical radiographs PISF collection (MMP‐8, TNF‐α)	*N* = 71 71 M *I*_c_ = 25 M *I*_v_ = 21M *C* = 25 M	*I*_c_ = 29.5 (5.8) *I*_v_ = 27.8 (3.1) *C* = 30.2 (4.4)	N/A	N/A	Peri‐implant mucositis (crestal bone loss < 3 mm on digital bitewing radiographs)	*I*_c_ = 8.3 (1.3) *I*_v_ = 4.1 (0.9)	*I*_c_ = 7.6 (3.3) *I*_v_ = 3.8 (1.5)
Al Deeb et al. (2020b), Saudi Arabia	Cohort study	PI, BoP, PD Radiographic analysis PISF collection (RANK‐L, OPG)	N = 75 71 M, 4 F I_c_ = 25 M I_v_ = 21 M, 4F C = 25 M	I_c_ = 44.7 (7.2) I_v_ = 35.6 (4.8) C = 41.3 (6.5)	N/A	N/A	Peri‐implantitis (“Peri‐implant diseases and conditions: Consensus report of workgroup 4 of the 2017 World Workshop on the Classification of Periodontal and Peri‐Implant Diseases and Conditions”—PD ≥ 6 mm, loss of bone≥ 3 mm apical of the most coronal portion of the intraosseous part of the implant	I_c_ = 12.6 (3.9) I_v_ = 4.1 (0.9)	I_c_ = 8.3 (2.1) I_v_ = 6.8 (2.5)
Al‐Hamoudi et al. (2020), Saudi Arabia	Cross‐sectional study	PI, PD, CAL, MBL, GI Radiographic analysis GCF, IL‐4, IL‐9, IL‐10, and IL‐13	N = 71 62 M, 9 F I_v_ = 32 M, 4F C = 30 M, 5 F	I_v_ = 47.7 (5.8) C = 46.5 (3.4)		Chronic moderate periodontitis (GI > 1, CAL ≥ 3 mm, PD ≥ 4 mm, and radiographic evidence of marginal bone loss (MBL) ≥ 3 mm below the cementoenamel junction (CEJ))		I_v_ = 17.6 (3.1)	I_v_ = 3.3 (0.5)
ALharthi et al. (2019), Country not specified	Cohort study	PI, BoP, CAL, PD, Missing teeth, PD ≥ 4 mm	*N* = 89 89 M I_c_ = 30 M I_v_ = 28M C = 31 M	I_c_ = 36.4 (2.8) I_v_ = 32.5 (4.8) C = 32.6 (3.5)		Periodontal inflammation (at least 6 sites with pockets of 4–8 mm) and Gingival inflammation (BoP≥ 30%)		I_c_ = 9.3 (4.6) I_v_ = 12.5 (0.8)	I_c_ = 10.4 (1.8) I_v_ = 3.1 (0.4)
Alhumaidan et al. (2022), Saudi Arabia	Cohort study	PD, GI, CAL, PI, MBL, whole salivary CL, IL‐1β, missing teeth, radiographic analysis	*N* = 54 36 M, 18 F I_c_ = 14 M, 4 F I_v_ = 12 M, 6F C = 10 M, 8 F	I_c_ = 45.6 (2.8) I_v_ = 41.3 (1.8) C = 42.2 (3.5)	Likely gingivitis from CAL and PD, but not specified			I_c_ = Not specified I_v_ = Not specified	I_c_ = 12.7 (2.2*) I_v_ = 6.8 (0.5)
AlJasser et al. (2021), Saudi Arabia	Case control study	PD, BoP, PI, MMP‐8, IL‐6, IL‐1β, and TIMP‐1	*N* = 60 31 M, 29 F I_c_ = 12 M, 8 F I_v_ = 9 M, 11F C = 10 M, 10 F	I_c_ = 54.1 I_v_ = 46.8 C = 46.9			Peri‐implantitis (“Classification of periodontal and peri‐implant diseases and conditions—2017, work group IV consensus report”—with ≥ 6 mm probing depth (ii) in tissue level implant bone level ≥ 3 mm apical either at smooth‐rough interface or implant coronal portion (iii) on gentle probing suppuration or bleeding initiates)	I_c_ = 9.2 (0.6) I_v_ = 6.5 (0.9)	I_c_ = 30* I_v_ = Not specified
Shah et al. (2023), United Kingdom	Cohort study	PD, CAL, PI, BoP, number of teeth present (excluding wisdom teeth), number of sites with PD≥ 5 mm	*N *= 220 90 M, implied 130 F I_c_ = 20 (11 M, 9 F) I_v_ = 20 (9 M, 11 F) C (NS) = 120 (48 M, 72 F) C(FS) = 60 (22 M, 38 F)	I_c_ = 44.1 (10.2) I_v_ = 47.8 (6.9) C (NS) = 48.0 (12.2) C (FS) = 49.0 (9.9)		Periodontal disease (“Periodontitis: consensus report of workgroup 2 of the 2017 World Workshop on the classification of periodontal and peri‐implant diseases and conditions”		I_c_ = 10.2 (6.4) I_v_ = Not specified C(FS) = Not specified	I_c_ = 20.0 (9.9) 10.7 (8.5*) I_v_ = 2.4 C(FS) = Not specified

Abbreviations: aPDT, antimicrobial photodynamic therapy; BoP, bleeding on probing; CAL, clinical attachment loss; C(FS), former smokers; CHx, chlorhexidine; C, Control Group (non‐smokers); GCF, gingival crevicular fluid; GI, gingival index; H_2_O_2_, hydrogen peroxide; IL, interleukin; I_c_, Intervention Group 1 (Cigarette use); I_v_, Intervention Group 2 (E‐cigarette use); MBL, marginal bone loss; MMP, matrix metalloproteinase; N/A, not applicable; OHI, oral hygiene instruction; PD, probing depth; PDT, photodynamic therapy; PI, plaque index; PISF, peri‐implant sulcus fluid; TIMP, tissue inhibitors of metalloproteinases; *, pack‐years.

### 3.2 Participant Disease Classification

Periodontal and peri‐implant diseases ranged across all studies (Table 1). A total of one study included participants with peri‐implant mucositis (*N* = 71), (Al Deeb et al. 2020a) two studies included participants with peri‐implantitis (*N* = 135) (Al Deeb et al. 2020b; AlJasser et al. 2021), two studies included participants with periodontitis (*N* = 291) (Al‐Hamoudi et al. 2020; Shah et al. 2023), one study reported patients to have “periodontal inflammation” (*N* = 89), (ALharthi et al. 2019), and one study did not specify the disease, (Alhumaidan et al. 2022), however, described gingivitis (*N* = 54) as per the 2017 Classification Scheme for Periodontal and Peri‐Implant Diseases and Conditions (Caton et al. 2018; Tonetti et al. 2018).

### 3.3 Therapies Used

Table 2 summarizes the therapies used by each study. Two studies, one concerning peri‐implantitis, and the other peri‐implant mucositis, performed full mouth mechanical debridement with a single session of photodynamic therapy with a diode laser and methylene blue photosensitizer [0.005%] (Al Deeb et al. 2020a, 2020b). They also provided participants with oral hygiene instruction and advised them to use chlorhexidine mouthwash (0.12%) (Al Deeb et al. 2020a, 2020b). Only one study for participants with peri‐implantitis provided surgical treatment (AlJasser et al. 2021). The study provided surgical open‐flap debridement and implant surface modification without grafting for implant sites unresponsive to nonsurgical periodontal therapy (NSPT) (AlJasser et al. 2021). Two studies aimed at treating periodontitis using forms of nonsurgical treatment (NSPT and professional‐mechanical‐plaque‐removal [PMPR]) involving ultrasonic scalers, hand curettes, and oral hygiene instruction (Alhumaidan et al. 2022; Shah et al. 2023). Four of the studies instructed participants to use chlorhexidine gluconate mouthwash as well (Al Deeb et al. 2020a, 2020b; Alhumaidan et al. 2022; AlJasser et al. 2021). One study aimed at treating “periodontal inflammation” provided full‐mouth ultrasonic scaling without root‐surface debridement using manual curettes, along with reinforced oral hygiene instruction (ALharthi et al. 2019). One study did not specify the disease it was treating and provided NSPT as the sole treatment modality (Alhumaidan et al. 2022).

**Table 2:** Study characteristics regarding therapy and adjunctive measures, baseline and post‐treatment outcomes, and re‐evaluation timelines.

Author (year)	Intervention and control	Adjunctive therapies	Parameters measured and baseline values, mean (SD)	Post‐treatment outcomes, mean (SD)	Re‐evaluation timeline and follow‐up and treatment outcomes
Al Deeb et al. (2020a)	I_c_ = Full‐mouth mechanical debridement (ultrasonic scaler) + PDT (diode laser and methylene blue photosensitizer) + OHI (toothbrushing) I_v_ = Full‐mouth mechanical debridement (ultrasonic scaler) + PDT (diode laser and methylene blue photosensitizer) + OHI (toothbrushing) C = Full‐mouth mechanical debridement (ultrasonic scaler) + PDT (diode laser and methylene blue photosensitizer) + OHI (toothbrushing)	All groups = CHx rinse 0.12% for 1 min 2/day for 14 days	PI (%): I_c_ = 45.3 (12.6) I_v_ = 37.6 (8.0) C = 32.4 (14.7) BoP (%): I_c_ = 13.8 (2.4) I_v_ = 18. (3.7) C = 37.4 (6.5) PD (mm): I_c_ = 4.1(0.8) I_v_ = 3.8 (0.6) C = 3.5 (0.4) TNF‐α (pg/ml): I_c_ = 482.35 (9.4) I_v_ = 397.66 (11.5) C = 269.58 (14.8) MMP‐8 (mg/ml): I_c_ = 308.48 (19.65) I_v_ = 286.81 (25.57) C = 242.74 (31.83)	PI (%): I_c_ = 13.2 (3.4) I_v_ = 17.4 (6.8) C = 15.8 (7.0) BoP (%): I_c_ = 21.6 (10.3) I_v_ = 24.7 (12.2) C = 12.8 (9.3) PD (mm): I_c_ = 3.7 (0.3) I_v_ = 3.2 (0.5) C = 2.7 (0.3) TNF‐α (pg/ml): I_c_ = 410.73 (19.13) I_v_ = 304.59 (12.27) C = 154.71 (19.82) MMP‐8 (mg/ml): I_c_ = 253.56 (23.24) I_v_ = 217.84 (36.78) C = 116.91 (17.61)	12‐week follow‐up All groups = PI statistically significant decrease from baseline (*p* < 0.001) I_c_ and I_v_ = BoP statistically significant increase from baseline for respective groups (*p* < 0.05) C = BoP statistically significant decrease from baseline (*p* < 0.001) All groups = PD no statistically significant differences I_c_ and I_v_ = MMP‐8 and TNF‐α statistically significant reduction from baseline (*p* < 0.05) C = MMP‐8 and TNF‐α statistically significant reduction from baseline (*p* < 0.001)
Al Deeb et al. (2020b)	I_c_ = Full‐mouth mechanical debridement (ultrasonic scaler) + PDT (diode laser and methylene blue photosensitizzer) + OHI (toothbrushing and flossing techniques) I_v_ = Full‐mouth mechanical debridement (ultrasonic scaler) + PDT (diode laser and methylene blue photosensitizer) + OHI (toothbrushing and flossing techniques) C = Full‐mouth mechanical debridement (ultrasonic scaler) + PDT (diode laser and methylene blue photosensitizer) + OHI (toothbrushing and flossing techniques)	All groups = CHx rinse 0.12% 2/daily for 1 min + avoid anti‐inflammatory medications	PI (%): I_c_ = 48.8 (11.4) I_v_ = 39.5 (9.2) C = 46.4 (12.3) BoP (%): I_c_ = 31.7 (6.2) I_v_ = 25.6 (5.8) C = 47.1 (16.3) PD (mm): I_c_ = 5.8 (1.7) I_v_ = 5.3 (1.5) C = 5.4 (0.9) RANK‐L (pg/ml): I_c_ = 813 (388) I_v_ = 726 (302) C = 789 (361) OPG (pg/ml): I_c_ = 1485 (794) I_v_ = 1349 (672) C = 1269 (648)	3‐month PI (%): I_c_ = 21.4 (6.8) I_v_ = 19.6 (8.5) C = 25.4 (7.8) BoP (%): I_c_ = 25.6 (3.5) I_v_ = 16.4 (5.2) C = 19.8 (4.3) PD (mm): I_c_ = 4.4 (1.4) I_v_ = 4.2 (1.6) C = 4.0 (1.4) RANK‐L (pg/ml): I_c_ = 683 (432) I_v_ = 527 (367) C = 343 (211) OPG (pg/ml): I_c_ = 1598 (1143) I_v_ = 1456 (1329) C = 1323 (1188) 6‐months PI (%): I_c_ = 16.1 (2.2) I_v_ = 14.5 (2.8) C = 18.2 (4.3) BoP (%): I_c_ = 20.4 (4.8) I_v_ = 14.9 (6.7) C = 18.5 (5.3) PD (mm): I_c_ = 3.7 (1.7) I_v_ = 3.3 (1.8) C = 3.0 (1.7) RANK‐L (pg/ml): I_c_ = 767 (562) I_v_ = 614 (501) C = 445 (324) OPG (pg/ml): I_c_ = 1364 (1032) I_v_ = 1271 (1137) C = 1173 (956)	3‐month and 6‐month follow‐ups I_v_ = PI statistically significant difference (less) than I_c_ and C at baseline (*p* < 0.05) All groups at both 3 and 6 months = PI significant decrease from baseline C = BoP statistically significant difference (more) than I_c_ and I_v_ at baseline (*p* < 0.05) I_c_ = BoP statistically significant difference between respective groups at 3‐months (*p* < 0.05) and significant difference compared to baseline at 6‐months, PD statistically significant difference between respective groups at 6‐months (*p* < 0.05) I_v_ and C = BoP significant difference compared to baseline (*p* < 0.05) All groups = PD significant difference compared with baseline; BoP significant difference compared with baseline I_v_ = RANK‐L statistically significant decrease from baseline at 3‐months (*p* < 0.05) C = RANK‐L statistically significant decrease from baseline at 3 and 6‐months (*p* < 0.05); statistically significant difference compared to I_c_ and I_v_ at 3 and 6 months (*p* < 0.05) All groups = no statistically significant difference at baseline or between groups at 3 and 6 months for OPG levels
Al‐Hamoudi et al. (2020)	I_v_ = SRP C = SRP	N/A	PI (%): I_v_ = 2.62 (0.43) C = 2.25 (0.16) GI: I_v_ = 0.36 (0.08) C = 2.55 (0.08) PD (mm): I_v_ = 5.2 (0.87) C = 5.0 (0.06) CAL (mm): I_v_ = 3.4 (0.2) C = 3.1 (0.2) MBL (mm): Mesial I_v_ = 4.8 (0.3) C = 4.7 (0.2) Distal I_v_ = 4.6 (0.2) C = 4.6 (0.1) GCF volume (µL): I_v_ = 1.65 (0.37) C = 1.74 (0.41) IL‐4 (pg/µL): I_v_ = 0.16 (0.12) C = 0.15 (0.13) IL‐9 (pg/µl): I_v_ = 0.33 (0.2) C = 0.5 (0.12) IL‐10 (pg/µl): I_v_ = 1.11 (0.08) C = 1.34 (0.15) IL‐13 (pg/µL): I_v_ = 0.85 (0.16) C = 0.74 (0.12)	PI (%): I_v_ = 1.91 (0.18) C = 0.62 (0.14) GI: I_v_ = 0.34 (0.06) C = 0.86 (0.1) PD (mm): I_v_ = 3.7 (0.2) C = 1.8 (0.07) CAL (mm): I_v_ = 3.2 (0.1) C = 2.7 (0.08) MBL (mm): Mesial I_v_ = 4.7 (0.08) C = 4.6 (0.03) Distal I_v_ = 4.5 (0.05) C = 4.5 (0.02) GCF volume (µl): I_v_ = 0.66 (0.14) C = 0.18 (0.09) IL‐4 (pg/µl): I_v_ = 0.71 (0.25) C = 1.52 (0.18) IL‐9 (pg/µl): I_v_ = 1.7 (0.21) C = 4.21 (0.3) IL‐10 (pg/µl): I_v_ = 3.15 (0.5) C = 6.63 (0.4) IL‐13 (pg/µl): I_v_ = 2.26 (0.21) C = 4.63 (0.47)	3‐month follow up I_v_ and C = PI and PD statistically significant decrease from baseline to 3‐months (*p* < 0.05) I_v_ = PI and PD statistically significant difference (more) compared to C at 3‐months (*p* < 0.05) I_v_ and C = No statistically significant difference between I_v_ and C in CAL and MBL at 3‐months All groups = MBL no statistical difference between baseline to 3‐months or between respective groups C = CAL statistically significant decrease from baseline to 3‐months (*p* < 0.05) I_v_ and C = GCF volume, IL‐4, IL‐9, IL‐10, and IL‐13 statistically significant difference from baseline to 3‐months (*p* < 0.05) I_v_ = GCF volume, IL‐4, IL‐9, IL‐10, and IL‐13 statistically significant difference compared to C at 3‐months (*p* < 0.05)
ALharthi et al. (2019)	I_c_ = Full‐mouth supragingival debridement (ultrasonic scaler) + OHI I_v_ = Full‐mouth supragingival debridement (ultrasonic scaler) + OHI C = Full‐mouth supragingival debridement (ultrasonic scaler) + OHI	N/A	PI (%): I_c_ = 49.4 (7.3) I_v_ = 43.5 (5.6) C = 46.3 (5.2) BoP (%): I_c_ = 17.2 (3.3) I_v_ = 11.6 (4.5) C = 38.2 (6.5) PD: I_c_ = 5.2 (0.4) I_v_ = 4.6 (0.2) C = 4.2 (0.3) CAL: I_c_ = 0 I_v_ = 0 C = 0 Number of sites with PD≥ 4 mm: I_c_ = 14.2 (1.5) I_v_ = 10.6 (1.2) C = 12.6 (2.2) Missing teeth: I_c_ = 5.2 (0.6) I_v_ = 4.5 (0.2) C = 4.8 (0.3)	3‐month: PI (%): I_c_ = 34.5 (4.6) I_v_ = 21.4 (2.8) C = 18.2 (1.6) BoP (%): I_c_ = 11.4 (2.4) I_v_ = 9.8 (0.3) C = 8.5 (0.4) PD: I_c_ = 4.4 (0.4) I_v_ = 3.1 (0.2) C = 2.7 (0.1) CAL: I_c_ = 0 I_v_ = 0 C = 0 Number of sites with PD≥ 4 mm: I_c_ = 7.1 (0.6) I_v_ = 0 C = 0 Missing teeth: I_c_ = 5.2 (0.6) I_v_ = 4.5 (0.2) C = 4.8 (0.3) 6‐month: PI (%): I_c_ = 38.6 (3.8) I_v_ = 19.4 (1.7) C = 20.3 (21.1) BoP (%): I_c_ = 13.5 (0.6) I_v_ = 10.2 (1.5) C = 10.4 (0.6) PD: I_c_ = 4.6 (0.1) I_v_ = 3.2 (0.4) C = 2.4 (0.2) CAL: I_c_ = 0 I_v_ = 0 C = 0 Number of sites with PD≥ 4 mm: I_c_ = 7.4 (0.5) I_v_ = 0 C = 0 Missing teeth: I_c_ = 5.2 (0.6) I_v_ = 4.5 (0.2) C = 4.8 (0.3)	3‐months and 6‐months follow‐ups I_c_ = statistically significant reduction at 3‐month follow‐up compared to baseline for PI, PD, and PD ≥ 4 mm (*p* < 0.05), no statistically significant difference in BoP at all time intervals I_v_ = statistically significant decrease in PI and PD at 3‐month and 6‐month (*p* < 0.05), no significant difference in BoP at all time intervals, no PD ≥ 4 mm at 3‐month and 6‐month follow‐up C = statistically significant reduction in PI, BoP, and PD at 3‐months and 6‐months (*p* < 0.05), no PD≥ 4 mm at 3‐months and 6‐months I_c_ compared to I_v_ = No significant difference at baseline for PI, BoP, PD, and PD≥ 4 mm. At 3‐month and 6‐month PI was statistically significantly higher in I_c_ compared to I_v_ (*p* < 0.05). At 6‐months PD was statistically significantly higher in I_c_ compared to I_v_ (*p* < 0.05). No significant difference between BoP for I_c_ and I_v_ at any time intervals. I_c_ had PD≥ 4 mm at 3‐month and 6‐month compared to I_v_ who did not. I_c_ compared to C = At baseline no significant difference in PI, PD, and PD≥ 4 mm; BoP statistically significantly higher in C compared to I_c_ at baseline (*p* < 0.01). At 3‐month and 6‐month PI and PD were statistically significantly higher in I_c_ compared to C (*p* < 0.05). No PD≥ 4 mm for C group at 3‐month and 6‐month. I_v_ compared to C = At baseline no significant differences in PI, PD, PD ≥ 4 mm; BoP statistically significantly higher in C compared to I_v_ at baseline (*p* < 0.01). At 3‐month and 6‐month no significant difference in PI, BoP, and PD. No PD≥ 4 mm at 3‐month and 6‐month follow‐up.
Alhumaidan et al. (2022)	I_c_ = NSPT + OHI I_v_ = NSPT + OHI C = NSPT + OHI	0.12% CHx mouth rinse 2/daily	PI (%): I_c_ = 2.1 (0.2) I_v_ = 1.8 (0.2) C = 2.1 (0.3) GI: I_c_ = 0.5 (0.07) I_v_ = 0.7 (0.05) C = 2.5 (0.2) PD (mm): I_c_ = 4.5 (0.3) I_v_ = 4.4 (0.5) C = 4.6 (0.5) CAL (mm): I_c_ = 1.7 (0.07) I_v_ = 1.4 (0.1) C = 1.4 (0.2) MBL (mm): Mesial I_c_ = 3.2 (0.4) I_v_ = 2.9 (0.5) C = 2.7 (0.4) Distal I_c_ = 3.3 (0.5) I_v_ = 3.04 (0.3) C = 2.7 (0.5) Missing teeth: I_c_ = 5.2 (0.4) I_v_ = 4.5 (0.1) C = 4.4 (0.5) Salivary flow rate (ml/min): I_c_ = 0.15 (0.03) I_v_ = 0.13 (0.01) C = 0.11 (0.02) IL‐1ß (pg/ml): I_c_ = 72.2 (9.3) I_v_ = 67.3 (5.1) C = 42.8 (3.7) Cortisol (pg/ml): I_c_ = 625.4 (204.8) I_v_ = 589.7 (153.3) C = 386.4 (87.5)	PI: I_c_ = 1.5 (0.3) I_v_ = 1.2 (0.04) C = 0.5 (0.007) GI: I_c_ = 0.5 (0.04) I_v_ = 0.5 (0.06) C = 0.4 (0.003) PD (mm): I_c_ = 2.5 (0.2) I_v_ = 2.6 (0.3) C = 0.6 (0.003) CAL (mm): I_c_ = 1.7 (0.04) I_v_ = 1.5 (0.08) C = 1.4 (0.06) MBL (mm): no radiographs taken at follow‐up Missing teeth: I_c_ = 5.2 (0.4) I_v_ = 4.5 (0.1) C = 4.4 (0.5) Salivary flow rate (ml/min): I_c_ = 0.13 (0.02) I_v_ = 0.12 (0.01) C = 0.12 (0.01) IL‐1ß (pg/ml): I_c_ = 54.1 (7.2) I_v_ = 60.7 (5.5) C = 6.4 (1.3) Cortisol (pg/ml): I_c_ = 516.8 (143.8) I_v_ = 422.8 (108.3) C = 141.8 (33.7)	12‐week follow‐up All groups = no statistically significant difference in CAL, PD, PI, and MBL at baseline I_v_ and I_c_ = statistically significantly higher gingival bleeding compared to C at baseline (*p* < 0.01), PI and PD were statistically significantly higher compared to C at 12‐weeks (*p* < 0.01) All groups = No statistically significant difference in GI and CAL at 12 = weeks I_v_ and I_c_ = No statistically significant difference in cortisol and IL‐1ß at baseline and 12‐weeks C = Statistically significant reduction in IL‐1ß and cortisol at 12‐weeks (*p* < 0.01)
AlJasser et al. (2021)	All groups = Open flap debridement + implant modification with bone recontouring (no bone grafting)	All groups received OHI. Depending on what peri‐implant pocket depth at maintenance visit adjunctive therapy of: no treatment, OHI (toothbrushing and interdental cleaning), local further debridement, re‐do of surgical resective surgery if needed, and/or topical antiseptic preparation of 3% H_2_0_2_ or 2% Chx. Use of systemic Abx (unclear who and which).	PD (mm): I_c_ = 6.9 (0.9) I_v_ = 6.3 (1.4) C = 6.5 (1.3) BoP (% of participants): I_c_ = 72 I_v_ = 76.5 C = 88.9 PI (% of participants): I_c_ = 55 I_v_ = 65 C = 100 MMP‐8 (pg/ng): I_c_ = 20.1 (6.3) I_v_ = 27.1 (5.3) C = 9.4 (4.3) IL‐6 (pg/ng): I_c_ = 153.7 (20.6) I_v_ = 183 (8.4) C = 47.2 (3.7) IL‐1ß (pg/ng): I_c_ = 3.55 (0.06) I_v_ = 3.62 (0.08) C = 2.84 (0.04) TIMP‐1 (pg/ng): I_c_ = 4.61 (3.4) I_v_ = 1.8 (2.4) C = 6.9 (2.0)	1‐month PD (mm): I_c_ = 4.4 (0.5) I_v_ = 4.7 (0.5) C = 4.2 (0.6) BoP (% of participants): I_c_ = 15 I_v_ = 10 C = 0 PI (% of participants): I_c_ = 20 I_v_ = 35 C = 0 MMP‐8 (pg/ng): I_c_ = 10.1 (1.6) I_v_ = 11.4 (1.8) C = 3.3 (0.8) IL‐6 (pg/ng): I_c_ = 63.2 (11.3) I_v_ = 85.6 (6.4) C = 15.6 (2.3) IL‐1ß (pg/ng): I_c_ = 1.96 (0.10) I_v_ = 2.14 (0.11) C = 1.28 (0.17) TIMP‐1 (pg/ng): I_c_ = 25.6 (16.8) I_v_ = 14.2 (4.8) C = 49.8 (19.6) 6‐month PD (mm): I_c_ = 4.7 (0.6) I_v_ = 5.5 (1.1) C = 4.5 (0.8) BoP (% of participants): I_c_ = 26.3 I_v_ = 47.4 C = 10.5 PI (% of participants): I_c_ = 21 I_v_ = 31.6 C = 5 MMP‐8 (pg/ng): I_c_ = 14.9 (16.3) I_v_ = 22.9 (35.2) C = 5.2 (4.6) IL‐6 (pg/ng): I_c_ = 65.4 (15.6) I_v_ = 96.3 (34.5) C = 18.6 (10.6) IL‐1ß (pg/ng): I_c_ = 2.01 (0.09) I_v_ = 2.44 (0.44) C = 1.32 (0.19) TIMP‐1 (pg/ng): I_c_ = 10.6 (5.4) I_v_ = 5.9 (4.1) C = 42.3 (25.6) 1‐year PD (mm): I_c_ = 4.8 (0.9) I_v_ = 5.6 (1.1) C = 4.4 (0.8) BoP (% of participants): I_c_ = 30 I_v_ = 50 C = 10 PI (% of participants): I_c_ = 20 I_v_ = 35 C = 5 MMP‐8 (pg/ng): I_c_ = 12.2 (5.6) I_v_ = 16.9 (7.9) C = 4.1 (2.2) IL‐6 (pg/ng): I_c_ = 80.6 (43.3) I_v_ = 120.6 (50) C = 19.4 (11.1) IL‐1ß (pg/ng): I_c_ = 2.3 (0.62) I_v_ = 2.84 (0.75) C = 1.47 (0.49) TIMP‐1 (pg/ng): I_c_ = 10.1 (5.8) I_v_ = 5.7 (4.1) C = 43.6 (24.4)	1‐month, 6‐months, 1‐year follow up C = TIMP‐1 significantly higher at all follow‐ups when compared to baseline I_v_ = PD increased after 6‐months and 1‐year, no statistically significant difference in mean values of MMP‐8 compared to I_c_ and C groups All groups = Significant change in PD, statistically significant difference in PI at 1‐month (*p* = 0.0016), statistically significant increase in BoP at 12‐months (*p* = 0.022), mean values for MMP‐8 at baseline significantly reduced compared to 1‐month—slight increase at 6‐months and 1‐year (no statistical significance), mean values of IL‐6 statistically significant reduction from baseline to 1‐month (*p* < 0.0001), statistically significant change in TIMP‐1 during all time intervals (*p* < 0.0001), significant increase in TIMP‐1 observed at 1‐year, increase in IL‐1ß at 1‐year but significantly lower than baseline Significant association between time periods and smoking type for IL‐6, IL‐1ß, and TIMP‐1 (*p* < 0.0001)
Shah et al. (2023)	All groups = professional‐ mechanical‐ plaque removal (supra‐ and subgingival ultrasonic scalars and hand curettes) + OHI (toothbrushing and interdental cleaning advice)	N/A	PI (%): I_c_ = 47.8 (21.6) I_v_ = 48.6 (21.4) C = 46.3 (19.1) C(FS) = 49.6 (21.2) BoP (%): I_c_ = 43.8 (23.6) I_v_ = 33.7 (26.1) C = 41.2 (23.1) C(FS) = 43.4 (27.3) PD (mm): I_c_ = 3.48 (1.03) I_v_ = 3.53 (0.95) C = 3.21 (0.78) C(FS) = 3.44 (0.95) CAL (mm): I_c_ = 4.17 (1.40) I_v_ = 4.42 (1.43) C = 3.72 (1.07) C(FS) = 3.92 (1.06) Number of teeth present (excluding wisdom teeth): I_c_ = 25.4 (2.2) I_v_ = 25.4 (3.2) C = 26.0 (2.5) C(FS) = 25.4 (2.7) Number of sites with PD ≥ 5 mm: I_c_ = 42.5 (29.8) I_v_ = 44.7 (30.3) C = 34.6 (23.2) C(FS) = 38.7 (25.3)	PI (%): I_c_ = 23.5 (12.2) I_v_ = 27.9 (24.0) C = 20.3 (12.3) C(FS) = 24.8 (17.3) BoP (%): I_c_ = 22.6 (18.4) I_v_ = 26.1 (19.7) C = 20.1 (13.8) C(FS) = 19.7 (14.3) PD (mm): I_c_ = 2.86 (0.89) I_v_ = 3.15 (0.74) C = 2.49 (0.56) C(FS) = 2.72 (0.59) CAL (mm): I_c_ = 3.74 (1.30) I_v_ = 4.31 (1.46) C = 3.18 (0.98) C(FS) = 3.52 (0.96) Number of teeth present (excluding wisdom teeth): I_c_ = 25.1 (2.6) I_v_ = 25.1 (3.4) C = 25.9 (2.6) C(FS) = 25.0 (2.8) Number of sites with PD≥ 5 mm: I_c_ = 25.0 (22.6) I_v_ = 32.2 (24.2) C = 14.9 (13.7) C(FS) = 20.0 (15.2)	6–12 weeks from last PMPR to reassessment, re‐evaluation differed (4, 5, 6, 7, or 8 months) follow‐up—multiple visits All groups = no statistically significant difference for PI, BoP, and number of teeth present (excluding wisdom teeth), statistically significant difference for PD (*p* < 0.0001), CAL (*p* < 0.0005), and number sites with PD ≥ 5 mm (*p* < 0.0005) at re‐evaluation I_v_ = least favorable treatment response when evaluating PD and CAL (*p* < 0.01), most minimal change in BoP at re‐evaluation I_v_ and I_c_ = no statistically significant difference in treatment response I_v_ and FS = outcome levels at follow‐up differed significantly for number of sites with PD ≥ 5 mm and mean PD

Abbreviations: Abx, antibiotics; aPDT, antimicrobial photodynamic therapy; BoP, bleeding on probing; CAL, clinical attachment loss; C(FS), former smokers; CHx, chlorhexidine; C, Control Group (non‐smokers); GCF, gingival crevicular fluid; GI, gingival index; H_2_O_2_, hydrogen peroxide; IL, interleukin; I_c_, Intervention Group 1 (Cigarette use); I_v_, Intervention Group 2 (E‐cigarette use); MBL, marginal bone loss; MMP, matrix metalloproteinase; N/A, not applicable; OHI, oral hygiene instruction; PD, probing depth; PDT, photodynamic therapy; PI, plaque index; PISF, peri‐implant sulcus fluid; TIMP, tissue inhibitors of metalloproteinases.

### 3.4 Quality Assessment of Included Studies

The NOS was selected and modified for assessment of cohort (*N* = 4), (Al Deeb et al. 2020b; ALharthi et al. 2019; Alhumaidan et al. 2022; Shah et al. 2023) case‐control (*N* = 1), (AlJasser et al. 2021) and cross‐sectional (*N* = 1) (Al‐Hamoudi et al. 2020) studies (Tables 3a, 3b, 3c). Five of the six studies received a quality rating of “good” as they adequately addressed the selection criteria, ensured comparability between groups, and clearly defined exposure measures (Al Deeb et al. 2020b; Al‐Hamoudi et al. 2020; ALharthi et al. 2019; Alhumaidan et al. 2022; AlJasser et al. 2021). One study received a quality rating of “fair” due to concerns regarding representativeness, as all e‐cigarette users were former conventional smokers, and the duration between cessation of conventional smoking and initiation of e‐cigarette use was not clearly defined, limiting the reliability of attributing the observed effects solely to e‐cigarette use (Shah et al. 2023). All five papers received a score of between 7 and 9, indicating high quality and low risk of bias (Al Deeb et al. 2020a, 2020b; Al‐Hamoudi et al. 2020; ALharthi et al. 2019; Alhumaidan et al. 2022; AlJasser et al. 2021; Shah et al. 2023). The Cochrane ROB‐2 tool was used for one randomized control trial study (Al Deeb et al. 2020a) of which received an overall rating of “some concerns,” due to the randomization process, deviation from intended intervention, missing outcome data, and measurements of the outcome and selection of the reported results, indicating a moderate risk of bias (Table 4) (Sterne et al. 2019).

**Table 3a:** Newcastle Ottawa Scale modified for cohort studies.

Study	Selection questions				Comparability question	Exposure questions			Quality
	Repressiveness of exposed cohort	Selection of non‐exposed	Ascertainment of exposure	Outcome/s not present at start	Comparability	Assessment of outcome	Adequate follow‐up length	Adequacy of follow up	
Al Deeb et al. (2020b)	*		*	*	**	*	*		Good
ALharthi et al. (2019) ALharthi et al. (2019)	*		*	*	**	*	*	*	Good
Alhumaidan et al. (2020) Alhumaidan et al. (2022)	*	*	*	*	**	*	*	*	Good
Shah et al. (2023)		*	*		**	*	*	*	Fair

**Table 3b:** Newcastle Ottawa Scale modified for case‐control studies.

Study	Selection Questions				Comparability Question	Exposure Questions			Quality
	Case definition	Representativeness	Selection of controls	Definition of controls	Comparability	Ascertainment of exposure	Method	Non‐response rate	
AlJasser et al. (2021) AlJasser et al. (2021)	*	*	*		**		*	*	Good

**Table 3c:** Newcastle Ottawa Scale modified for cross‐sectional studies.

Study	Selection Questions				Comparability Question	Exposure Questions		Quality
	Representativeness of the case	Sample size	Non‐response rate	Ascertainment of screening tool	Potential co‐founders	Assessment of outcome	Statistical test	
Al‐Hamoudi et al. (2020)	*	*		*	*	**	*	Good

*Note:* Thresholds for converting the Newcastle–Ottawa Scales to AHRQ standards (good, fair, and poor) (Wells et al. 2024a, 2024b). Good quality = 3 or 4 stars in select domain AND 1 or 2 stars in comparability domain AND 2 or 3 stars in outcomes/exposure domain. Fair quality = 2 stars in selection domain AND 1 or 2 stars in comparability domain AND 2 or 3 stars in outcome/exposure domain. Poor quality = 0 or 1 star in selection domain OR 0 stars in comparability OR 0 or 1 stars in outcome/exposure domain.

**Table 4:** Cochrane Risk of Bias (ROB) tool.

Study	Randomization process	Deviations from the intended interventions	Missing outcome data	Measurements of the outcome	Selection of the reported result	Overall
(Al Deeb et al. (2020a))	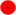	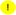	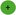	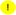	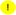	Some concerns

*Note:* 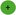 Low risk; 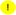 Some concerns; 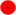 High risk.

### 3.5 Clinical Periodontal and Peri‐Implant Parameters

#### 3.5.1 Plaque Index (PI)

All seven studies analyzed PI (Table 2) (Al Deeb et al. 2020a, 2020b; Al‐Hamoudi et al. 2020; ALharthi et al. 2019; Alhumaidan et al. 2022; AlJasser et al. 2021; Shah et al. 2023). In patients with peri‐implant disease, there was a significant reduction in PI among all three groups (cigarette smokers, vape users, and non‐smokers) from baseline to 3 and 6‐month follow‐up (Al Deeb et al. 2020b). Similarly, another study found a statistically significant reduction in PI among all three groups from baseline to 3‐month follow‐up for patients with peri‐implant disease receiving PDT (Al Deeb et al. 2020a). The vape user group had the lowest PI scores at all time points (Al Deeb et al. 2020b). Plaque index for vape users decreased at a similar rate to cigarette smokers with peri‐implantitis from baseline to 1 and 6‐months follow up, however, PI was the highest for vape users 1‐year post treatment in comparison to cigarette users and non‐smokers (AlJasser et al. 2021). Among peri‐implant mucositis patients, PI was found to be higher in cigarette smokers when compared to vapers and non‐smokers at baseline (Al Deeb et al. 2020a). However, plaque index was found to be highest after treatment among the vape user group (Al Deeb et al. 2020a). In patients with periodontitis, PI scores were higher among participants with periodontitis who vape in comparison to non‐smokers at both baseline and 3 months post‐treatment (Al‐Hamoudi et al. 2020). There was a statistically significant difference in PI between vape users and non‐smokers at 3‐month follow‐up, with vape users exhibiting higher PI scores (Al‐Hamoudi et al. 2020). Plaque index was similar for all participants with periodontitis (cigarette smokers, non‐smokers, former smokers) at baseline; however, post‐treatment, vape users had the highest PI and the least overall change in PI compared to baseline value (Shah et al. 2023). Another study found PI of participants with periodontal inflammation to have a statistically significant decrease in PI for cigarette, vape, and non‐smokers; however, cigarette smokers maintained a significantly higher PI in comparison to vape users and non‐smokers at both 3 and 6 months post‐treatment (ALharthi et al. 2019). Plaque index was statistically higher in vape and cigarette users, in comparison to non‐smokers with gingivitis post‐treatment; however, cigarette smokers maintained a slightly higher PI than vape users from baseline to 12‐week follow‐up (Alhumaidan et al. 2022).

#### 3.5.2 Bleeding on Probing (BoP)

Bleeding on probing was reported as a parameter in five studies (Table 2) (Al Deeb et al. 2020a, 2020b; ALharthi et al. 2019; AlJasser et al. 2021; Shah et al. 2023). One study assessing participants with peri‐implant mucositis found significantly lower BoP scores for both cigarette smokers and vape users in comparison to non‐smokers at baseline; however, a statistically significant increase was seen in BoP scores for both respective groups at 12‐week follow‐up, whereas non‐smokers had a statistically significant decrease post‐treatment (Al Deeb et al. 2020a). Among peri‐implantitis patients, BoP was the lowest for vape users at baseline, 3‐months, and 6‐months follow‐up (Al Deeb et al. 2020b). However, BoP was found to be significantly lower for both non‐smokers and vape users at 3‐months post‐treatment in comparison to cigarette users (Al Deeb et al. 2020b). For peri‐implantitis patients, all groups had a significant decrease in BoP from their respective baseline, but no statistically significant difference was found between groups at 6‐months post‐treatment (Al Deeb et al. 2020b). Another study assessing peri‐implantitis patients recorded BoP scores as a binary system (yes/no), which found that BoP decreased 1‐year post‐treatment compared to baseline; however, it had a statistically significant increase from 1‐month post‐treatment to 1‐year post‐treatment (AlJasser et al. 2021). In patients with periodontitis, there is no statistically significant difference in BoP at baseline between vape and cigarette users; however, there was a statistically significant difference between vape users and non‐smokers at baseline, with non‐smokers presenting with higher BoP scores (ALharthi et al. 2019). Both vape users and cigarette smokers had no significant difference in BoP at all time intervals; however, only non‐smokers exhibited a statistically significant decrease in BoP post‐treatment (ALharthi et al. 2019). For periodontitis patients, BoP scores were lowest for vape users compared to cigarette users, non‐smokers, and former smokers at baseline. They exhibited the least change in BoP score at follow‐up (Shah et al. 2023).

#### 3.5.3 Probing Depth (PPD)

Probing depth was reported in all seven studies (Table 2) (Al Deeb et al. 2020a, 2020b; Al‐Hamoudi et al. 2020; ALharthi et al. 2019; Alhumaidan et al. 2022; AlJasser et al. 2021; Shah et al. 2023). In patients with “periodontal inflammation”, one study reported a statistically significant decrease in PPD at both 3‐ and 6‐months following supragingival debridement and OHI in vapers (ALharthi et al. 2019). The authors reported that PPD was higher among cigarette users when compared to vape users following supragingival debridement and OHI at all periods (ALharthi et al. 2019). In addition, vape users had a higher PPD in comparison to non‐smokers at all periods (ALharthi et al. 2019).

Two studies found that both vape and cigarette users maintained similarly high PPD in comparison to non‐smokers post‐treatment following NSPT and PMPR, both with OHI for participants with gingivitis and periodontitis, respectively (Alhumaidan et al. 2022; Shah et al. 2023). One study found that both vape and cigarette users had PPD that was statistically significantly higher than non‐smokers at the 12‐week follow‐up (Alhumaidan et al. 2022). The other study found a statistically significant difference for PPD between all groups at follow‐up, with vape users exhibiting the least favorable treatment response, with the least PPD reduction compared to all other groups (Shah et al. 2023). For periodontitis, there was a statistically significant reduction in PPD for vape users following SRP from baseline to 3‐months post‐treatment; this was statistically significantly worse in comparison to non‐smokers post‐treatment (Al‐Hamoudi et al. 2020).

There was no statistical significance between the groups after therapy in peri‐implant mucositis patients (Al Deeb et al. 2020a). In peri‐implantitis patients, one study showed a significant reduction in PPD in vape users and cigarette smokers at 6‐months following antimicrobial PDT, with only cigarette users showing a statistical significance (Al Deeb et al. 2020b). However, cigarette users had a significantly higher PPD compared to non‐smokers and vape users 6‐months post‐treatment (Al Deeb et al. 2020b). Non‐smokers and vape users did not respond differently in this study (Al Deeb et al. 2020b). PPD was found to be highest in cigarette users at baseline in comparison to vape users and non‐smokers for patients with peri‐implantitis (Al Deeb et al. 2020b). However, another study found that vape users maintained the highest residual PPD at all periods post‐treatment in comparison to cigarette users and non‐smokers with peri‐implantitis (AlJasser et al. 2021).

#### 3.5.4 Clinical Attachment Loss

Four of the seven studies included clinical attachment loss (CAL) (Table 2) (Al‐Hamoudi et al. 2020; ALharthi et al. 2019; Alhumaidan et al. 2022; Shah et al. 2023). Three of which included participants with periodontitis (Al‐Hamoudi et al. 2020; ALharthi et al. 2019; Shah et al. 2023). Al‐Hamoudi et al. found no statistically significant difference in CAL between vape users and non‐smokers (Al‐Hamoudi et al. 2020). One study reported a statistically significant difference in CAL across all groups post‐treatment compared to their baseline (Shah et al. 2023). Vape users had the least favorable response to treatment when compared to cigarette users, former smokers, and non‐smokers with periodontitis, whereby CAL was statistically significantly higher at all periods (Shah et al. 2023). Alhumaidan et al. included CAL when analyzing gingivitis and reported no changes in CAL 12 weeks post‐treatment for cigarette, vape users, or non‐smokers (Alhumaidan et al. 2022). ALharthi et al. analyzed “periodontal inflammation” and had values of zero for both baseline and post‐treatment in all groups (ALharthi et al. 2019).

#### 3.5.5 Marginal Bone Loss (MBL)

Marginal bone loss was included in two studies (Table 2) (Al‐Hamoudi et al. 2020; Alhumaidan et al. 2022). There was no statistically significant difference post‐treatment for vape users and non‐smokers for patients with periodontitis who received SRP (Al‐Hamoudi et al. 2020). One study did not report any data for MBL at follow‐up as radiographs were only taken at baseline (Alhumaidan et al. 2022).

#### 3.5.6 Tooth Loss/Missing Teeth

Two studies analyzed the number of teeth missing (ALharthi et al. 2019; Alhumaidan et al. 2022), whereas one study documented the number of teeth present (Table 2) (Shah et al. 2023). All three reported no significant difference between groups for the number of missing/present teeth at any follow‐up period for both gingivitis or periodontitis patients (ALharthi et al. 2019; Alhumaidan et al. 2022; Shah et al. 2023).

### 3.6 The Impact of Treatment on Biological Parameters

#### 3.6.1 TNF‐α

One of the seven studies analyzed Tumor Necrosis Factor‐alpha (TNF‐α) in peri‐implant sulcular fluid (PISF) from patients with peri‐implant mucositis (Table 2) (Al Deeb et al. 2020a). There was a statistically significant reduction in TNF‐α for cigarette users, vape users, and non‐smokers in comparison to their baseline levels (Al Deeb et al. 2020a). No group comparisons were explicitly made; however, the tables indicated higher TNF‐α counts in cigarette smokers than in vape users at both baseline and 12‐week follow‐up, and both cigarette and vape users had consistently higher TNF‐α values than those of non‐smokers (Al Deeb et al. 2020a). One study mentioned TNF‐α in its aim; however, no further mention was made in the results or discussion (AlJasser et al. 2021).

#### 3.6.2 MMP‐8

Two of the seven included studies analyzed metalloproteinase‐8 (MMP‐8) in PISF and in a saliva sample (Table 2) (Al Deeb et al. 2020a; AlJasser et al. 2021). For peri‐implant mucositis, Al Deeb et al. found a statistically significant reduction in MMP‐8 for cigarette users, vape users, and non‐smokers at 12‐weeks after therapy (Al Deeb et al. 2020a). No significant difference was reported between groups; however, the non‐smokers had the lowest MMP‐8 values and cigarette users the highest (Al Deeb et al. 2020a). For peri‐implantitis, AlJasser et al. reported a significant reduction after treatment in MMP‐8 at the 1‐month follow‐up for cigarette users, vape users, and non‐smokers (AlJasser et al. 2021). Despite no statistically significant differences in MMP‐8 across all groups and their baselines at 1‐month, 6‐months, and 1‐year, vape users had notably higher MMP‐8 values at each time point than smokers and non‐smokers (AlJasser et al. 2021).

#### 3.6.3 RANKL

One study analyzed the receptor activator of nuclear factor Kappa‐β Ligand (RANKL) in PISF fluid from patients with peri‐implantitis (Table 2) (Al Deeb et al. 2020b). The study performed a 3‐month and 6‐month follow‐up (Al Deeb et al. 2020b). The non‐smokers had a statistically significant difference in RANKL at both time periods compared to cigarette and vape users, where there was a statistically significant decline in RANKL for non‐smokers at both 3 and 6 months post‐treatment (Al Deeb et al. 2020b). Vape users experienced a statistically significant decrease in RANKL at the 3‐month follow‐up, but there was an increase at the 6‐month follow‐up (Al Deeb et al. 2020b). While cigarette smokers had the highest values of RANKL markers across all time periods, at baseline, the vape users had lower RANKL values than non‐smokers, but by months 3 and 6, they were higher than non‐smokers (Al Deeb et al. 2020b).

#### 3.6.4 OPG

One of the seven studies included an analysis of osteoprotegerin (OPG) in PISF for patients with peri‐implantitis (Table 2) (Al Deeb et al. 2020b). For all groups (cigarette smokers, vape users, and non‐smokers), while not statistically significant, there was a slight increase in OPG in PISF at the 3‐month follow‐up, which then reduced at the 6‐month follow‐up (Al Deeb et al. 2020b). Cigarette smokers had the highest OPG values that consistently decreased from baseline to 6‐months; however, it was noted that OPG levels increased from baseline at the 3‐month mark for vape users and non‐smokers before decreasing again at the 6‐month mark (Al Deeb et al. 2020b).

#### 3.6.5 Interleukins

Three of the seven studies analyzed included interleukin proteins collected from GCF, (Al‐Hamoudi et al. 2020; Alhumaidan et al. 2022) and saliva (Table 2) (AlJasser et al. 2021). One study analyzed IL‐4, IL‐9, IL‐10, and IL‐13 levels among periodontitis patients and found no statistical difference at baseline between vape users and non‐smokers (Al‐Hamoudi et al. 2020). However, 3‐months post‐treatment, vape users had statistically significantly lower values of IL‐4, IL‐9, IL‐10, and IL‐13 compared to non‐smokers, with statistically significant differences from respective baseline values for all interleukins (Al‐Hamoudi et al. 2020). Two studies examined IL‐1β (Alhumaidan et al. 2022; AlJasser et al. 2021). In participants with gingivitis, there was no significant difference in IL‐1β levels at baseline for cigarette smokers, vape users, and non‐smokers (Alhumaidan et al. 2022). However, IL‐1β was the highest among vape users 12‐weeks post‐treatment (Alhumaidan et al. 2022). Only non‐smokers showed a statistically significant reduction in IL‐1β 12 weeks post‐treatment (Alhumaidan et al. 2022). One study analyzing peri‐implantitis patients found a statistically significant change in IL‐1β for all groups (vape users, cigarette smokers, non‐smokers) across all periods (baseline, 1‐month, 6‐months, and 1‐year) (AlJasser et al. 2021). IL‐1β values were the highest for vape users at all periods, the lowest being non‐smokers (AlJasser et al. 2021). There was a statistically significant reduction in IL‐6 for cigarette smokers, vape users, and non‐smokers from baseline to 1‐month (AlJasser et al. 2021). Vape users had consistently higher levels of IL‐6 compared to cigarette users and non‐smokers at any time (baseline, 1‐month, 6‐months, and 1‐year); however, these values were not statistically significant (AlJasser et al. 2021). The researchers reported a statistically significant association between periods and smoking type for IL‐6 and IL‐1ß (AlJasser et al. 2021).

#### 3.6.6 TIMP‐1

One study analyzed TIMP‐1 for patients with peri‐implantitis (Table 2) (AlJasser et al. 2021). There was a statistically significant change in TIMP‐1 for all groups (vape users, cigarette users, and non‐smokers) during all periods (1‐month, 6‐months, and 1‐year) (AlJasser et al. 2021). Only non‐smokers showed a significant increase in TIMP‐1 at follow‐up compared to baseline. In contrast, cigarette and vape users did not (AlJasser et al. 2021). Vape users demonstrated the lowest TIMP‐1 markers at all time frames, values notably lower than those of cigarette smokers, and non‐smokers had a significantly higher TIMP‐1 value at all time intervals (AlJasser et al. 2021). The authors did not make comparisons between the groups; however, they found a statistically significant association between time and smoking type for TIMP‐1 (AlJasser et al. 2021).

#### 3.6.7 Salivary Flow Rate

One study analyzed salivary flow rate for participants with gingivitis (Table 2) (Alhumaidan et al. 2022). There was no difference in flow rate for cigarette users, vape users, or non‐smokers, at baseline or follow‐up (Alhumaidan et al. 2022).

#### 3.6.8 GCF

One study analyzed GCF, for periodontitis patients (Table 2) (Al‐Hamoudi et al. 2020). At baseline, there was no significant difference in GCF volume for vape users and non‐smokers (Al‐Hamoudi et al. 2020). At 12 weeks post‐treatment, there was a statistically significant reduction in GCF volume for both respective groups (non‐smokers and vape users) (Al‐Hamoudi et al. 2020). However, post‐treatment vape users had a statistically significantly higher GCF volume compared to non‐smokers (Al‐Hamoudi et al. 2020).

#### 3.6.9 Cortisol

One study analyzed salivary cortisol levels among patients with gingivitis (Table 2) (Alhumaidan et al. 2022). Non‐smokers had a statistically significant reduction in cortisol levels 12 weeks post‐treatment; however, there was no significant decline in cortisol levels for cigarette smokers and vape users when compared to their baseline values and 12 weeks post‐treatment (Alhumaidan et al. 2022). Vape users continued to demonstrate high cortisol levels comparable to those of cigarette users in whole saliva at 12‐week follow‐up (Alhumaidan et al. 2022).

### 3.7 Follow‐Up

All studies included baseline parameters, which were compared to various follow‐up intervals (Table 2) (Al Deeb et al. 2020a, 2020b; Al‐Hamoudi et al. 2020; ALharthi et al. 2019; Alhumaidan et al. 2022; AlJasser et al. 2021; Shah et al. 2023). Five studies reported follow‐up data at 12‐weeks/3‐months only, (Al Deeb et al. 2020a; Al‐Hamoudi et al. 2020; Alhumaidan et al. 2022) two studies reported follow‐up data at both 3‐ and 6‐months (Al Deeb et al. 2020b; ALharthi et al. 2019) and one study reported follow‐up data at 1 month, 6 months, and 1 year post‐treatment (AlJasser et al. 2021). One study reported retrospective data over a 24‐month period, with intervals ranging from 4 to 8 months (Shah et al. 2023).

### 3.8 Adverse Effects/Complications Associated With Treatment

Of all the included studies, Al Deeb et al. was the only study that reported adverse effects or complications related to periodontal or peri‐implant therapies (Al Deeb et al. 2020a). Al Deeb et al. observed that patients treated with PDT and MD experienced transient bleeding, which was attributed to PDT minimizing the deleterious effects of nicotine at the gingival level (Al Deeb et al. 2020a).

## 4 Discussion

To the best of our knowledge, this is the first systematic review to analyze the impact of vaping on the effectiveness of periodontal and peri‐implant therapy by examining both clinical parameters and immunological markers and comparing the results to those of control groups (cigarette smokers and non‐smokers). Our results showed that vape users have worse periodontal and peri‐implant clinical parameters and higher concentrations of pro‐inflammatory biomarkers than non‐smokers, following similar trends to those of cigarette smokers. This study offers new insights by exploring the relationship between vaping and treatment outcomes and therapeutic response. However, due to heterogeneity among the included studies in study design, outcome measures, exposure definitions, and follow‐up periods, a meta‐analysis could not be performed. Consequently, the interpretation of the results is limited, as conclusions were drawn from consistent trends observed across studies rather than from pooled quantitative estimates; therefore, these findings should be interpreted with caution.

Six of the seven studies have used NSPT, ranging from full‐mouth mechanical debridement to supragingival and root‐surface debridement, using ultrasonic or mechanical instrumentation (Al Deeb et al. 2020a, 2020b; Al‐Hamoudi et al. 2020; ALharthi et al. 2019; Alhumaidan et al. 2022; Shah et al. 2023). One study utilized surgical open‐flap debridement after unsuccessful therapy with NSPT (AlJasser et al. 2021). NSPT remains the gold standard for periodontal treatment (Herrera et al. 2022; Sanz et al. 2012, 2020). It has been demonstrated that cigarette smokers have poorer periodontal outcomes than non‐smokers following NSPT and surgical periodontal therapy (SPT), (Chang et al. 2021) due to reduced tissue healing capacity (Darby et al. 2005), vasoconstriction, inflammation (Scheres et al. 2010), oxidative stress (Lütfioğlu et al. 2021), and altered oral microbiome (Thiem et al. 2023). The long‐term effects of vapes on periodontal treatment outcomes are not well studied; however, many electronic cigarettes contain nicotine and other harmful substances, such as propylene glycol and flavorings, which can produce local vasoconstriction, increase pro‐inflammatory mediators, and reduce blood flow to negatively impact periodontal healing (Thiem et al. 2023; Beklen and Uckan 2021; Goniewicz et al. 2014; Spindle et al. 2018). In addition, other authors highlighted that vape use negatively impacts peri‐implant health by increasing oxidative stress, DNA damage, and inflammatory reactions (Javed, BinShabaib, et al. 2017; Lerner et al. 2015). Vaping has been associated with increased probing depth, bone loss, bone destruction mediators in peri‐implant tissues, and decreased BoP (Guney et al. 2024). Similarly, nicotine has detrimental effects on peri‐implant health, including impaired cellular healing response and increased biofilm colonization on titanium implants (Barão et al. 2015; Ghanem et al. 2017). Corroborating, our results show that BoP for vape users followed similar trends as cigarette users, both pre‐ and post‐treatment (Al Deeb et al. 2020a, 2020b; ALharthi et al. 2019; Shah et al. 2023). This may be attributed to the similar vasoconstrictive effects of nicotine and other toxic chemicals found in electronic cigarettes.

Our results show that post‐treatment vape users responded similarly to cigarette smokers with poorer overall outcomes than non‐smokers. Previous studies have demonstrated that cigarette smokers present poorer peri‐implant and periodontal outcomes, more severe periodontal parameters, and higher levels of pro‐inflammatory markers than non‐smokers (Alwithanani 2023; Casado et al. 2019; Leite et al. 2018; Vámos et al. 2024). This systematic review has found that vape users, whilst not as severe in most parameters, have similar clinical parameters to those of cigarette smokers and worse clinical outcomes than non‐smokers at both baseline and post‐treatment (Al Deeb et al. 2020a; ALharthi et al. 2019; Alhumaidan et al. 2022; AlJasser et al. 2021; Shah et al. 2023). Numerous studies have demonstrated higher levels of plaque index, pocket depth, clinical attachment loss, and marginal bone loss amongst vape users in comparison to non‐smokers (Al‐Aali et al. 2018a; Alqahtani et al. 2019; AlQahtani et al. 2018; ArRejaie et al. 2019; BinShabaib et al. 2019; Javed, Abduljabbar, et al. 2017; Mokeem et al. 2018). Corroborating our results shows that vape users and cigarette smokers both have consistently higher PI than non‐smokers following periodontal and peri‐implant therapy (Al Deeb et al. 2020a; Alhumaidan et al. 2022; AlJasser et al. 2021; Shah et al. 2023). The majority of included studies focused on oral hygiene reinforcement; however, two did not (Al‐Hamoudi et al. 2020; AlJasser et al. 2021). Plaque accumulation is a major etiological factor in periodontal and peri‐implant diseases; therefore, meticulous oral hygiene practices are recommended (Herrera et al. 2023; Nazir 2017). As part of comprehensive periodontal and peri‐implant treatment, preventive measures such as oral hygiene instruction should be provided to the patient routinely. Thus, shorter supportive periodontal care appointments may be recommended for both vapers and smokers. It is worth noting that advice on smoking cessation was not provided in any of the included studies.

In addition, PPD was found to be higher among vape users in comparison to non‐smokers following treatment, (Al‐Hamoudi et al. 2020; ALharthi et al. 2019; Alhumaidan et al. 2022) where some studies found the least PPD improvement amongst vape users when compared to both cigarette smokers and non‐smokers (AlJasser et al. 2021; Shah et al. 2023). Probing depths are consistently higher amongst cigarette users compared to non‐smokers, with vape users following a similar trend. This may result from prolonged exposure to harmful chemicals, which affect blood flow and impair periodontal healing after therapy (Thiem et al. 2023; Beklen and Uckan 2021; Goniewicz et al. 2014; Spindle et al. 2018). In addition, the poorer periodontal outcomes in vapers compared with non‐smokers emerge with relative consistency despite methodological differences. In contrast, probing depth changes and biomarker profiles vary substantially across studies and therefore warrant more cautious interpretation.

The adverse effects of conventional cigarette smoking on the host immune response have been sufficiently linked to the activation of inflammatory processes, cytokine production, and periodontal/peri‐implant tissue destruction (Pesce et al. 2022). In contrast to cigarette smokers, the effect of e‐cigarettes on oral cytokine fluids has been scarcely studied in the literature (D'Ambrosio et al. 2022). Studies have shown higher levels of PGE2 and IL‐1β in cigarette smokers over vape users in periodontal disease patients (Verma et al. 2021). On the other hand, one study included in this systematic review found IL‐1β to be the highest post‐treatment in patients with gingivitis (Alhumaidan et al. 2022), and another at all follow‐up appointments in patients with peri‐implantitis (AlJasser et al. 2021). IL‐1β is a potent stimulator of periodontal tissue destruction, as it promotes bone resorption and production of tissue‐degrading proteinases (Chmielewski and Pilloni 2023; Ng et al. 2007; Rocha et al. 2014; Tobón‐Arroyave et al. 2008).

Only one study included in this systematic review evaluated GCF volume, showing that vape users had a statistically significantly higher GCF volume compared to non‐smokers after therapy (Al‐Hamoudi et al. 2020). Gingival crevicular fluid acts as a medium for inflammatory mediators, and an increase in GCF volume may indicate periodontal inflammation (Bevilacqua et al. 2016). One included study found higher salivary cortisol levels among cigarette smokers and vape users compared to non‐smokers (Alhumaidan et al. 2022). Higher serum and salivary cortisol levels have been linked to more severe periodontal parameters, such as BoP, CAL, and PD (Ishisaka et al. 2008, 2007). A correlation between psychological stress and increased quantities of pro‐inflammatory cytokines (IL‐1β, IL‐6, IL‐8) in GCF has also been found in the literature (Giannopoulou et al. 2003). One of the studies included in this review, assessing patients with peri‐implantitis, found that vape users had high levels of IL‐6, IL‐1β, and MMP‐8 at all periods pre‐ and post‐treatment (AlJasser et al. 2021), and another with high levels of IL‐1β in gingivitis patients post‐treatment (Alhumaidan et al. 2022). A 10‐year retrospective study found that inflammation around teeth and implants may be linked to increased levels of MMP‐8 and IL‐1β in PISF or GCF (Ramseier et al. 2016). Cytokine IL‐1β has been demonstrated to be present in increased quantities within GCF in patients with periodontitis, with an increased concentration of IL‐1β correlating with increased disease severity (Engebretson et al. 2002; Konopka et al. 2012; Zhong et al. 2007). Another study included in this review found high levels of TNF‐α in vape and cigarette users in comparison to non‐smokers in peri‐implant disease (Al Deeb et al. 2020a). This is consistent with the current body of literature and may suggest increased inflammation and destruction. TNF‐α, IL‐6, and MMP‐8 have been suggested to have a role in accelerating peri‐implant tissue destruction, resulting in alveolar bone loss around dental implants, and there may be higher levels of these inflammatory markers for those who use e‐cigarettes, further increasing their risk of peri‐implant disease (Duarte et al. 2016; Teixeira Neves et al. 2022). Interestingly, one study found that non‐smokers had a statistically, significantly higher level of IL‐4, IL‐9, IL‐10, and IL‐13 in comparison to vape users at 3‐months post‐treatment (Al‐Hamoudi et al. 2020). These regulatory cytokines (IL‐4, IL‐10, and IL‐13) have anti‐inflammatory properties and regulate the human immune response by protecting the host from a hyper‐inflammatory pathogenic response (Duarte et al. 2015; Opal and DePalo 2000; Saraiva et al. 2020). Cytokine IL‐10 function has been demonstrated to reduce disease activity in periodontal diseases (Neurath and Kesting 2024; Ramadan et al. 2020), and cytokines IL‐4, IL‐10, and IL‐13 play an important role in inhibiting bone resorption (Duarte et al. 2015; Cetinkaya et al. 2013; Hong et al. 2024). A landmark study conducted by Ganesan et al. analyzed the effects of e‐cigarette smokers' cytokine expression in GCF in periodontally healthy individuals and found that e‐cigarette users had significantly higher amounts of IL‐2, IL‐6, granulocyte‐macrophage colony‐stimulating factor (GM‐CSF), TNF‐α, and interferon‐gamma (IFN‐*γ*), and lower levels of IL‐10 in comparison to non‐smokers (Ganesan et al. 2020). In addition, the authors found that while e‐cigarettes and conventional cigarettes were associated with higher levels of inflammation, they were mediated via different pathways (Ganesan et al. 2020). Cytokine IL‐6 has been strongly linked to the inflammatory processes in periodontal diseases, especially for its role in regulating T‐cell subsets, activation of IL‐17 secreting T‐helper cells, increasing osteoclastic activity, and signaling an acute‐phase response, for example, C‐reactive proteins (Mazurek‐Mochol et al. 2024). While IL‐6 is an important cytokine involved in the host response to bacterial infections, various other cytokines such as TNF‐α are produced along with the activation of MMPs, ultimately resulting in the breakdown of the extracellular matrix (Neurath and Kesting 2024; Isola et al. 2021; Borilova Linhartova et al. 2020; Evangeliou et al. 2021; Rudick et al. 2019; Apolinário Vieira et al. 2021; Bhavsar et al. 2019). Hence, the higher levels of these pro‐inflammatory cytokines in vapers compared to non‐smokers may be associated with an increased risk of disease progression. Tissue inhibitors of metalloproteinases (TIMPs) play a role in balancing and regulating MMP activity (de Brouwer et al. 2022). Tissue‐inhibitors of metalloproteinase‐1 (TIMP‐1) is not a reliable measure of periodontal disease in saliva collection (de Brouwer et al. 2022; Figueiredo et al. 2020), however, TIMP‐1 to MMP ratio has been found to display higher levels in peri‐implantitis cases (Lumbikananda et al. 2024). Osteoclast differentiation and bone resorption are stimulated by RANKL (Belibasakis and Bostanci 2012). When OPG binds to RANKL bone resorption is down‐regulated (Belibasakis and Bostanci 2012). Vape users with peri‐implantitis initially had the lowest RANKL markers; however, they demonstrated higher values than non‐smokers following treatment (Al Deeb et al. 2020b). Values of OPG for vape users increased shortly after treatment; however, they decreased by 6‐months (Al Deeb et al. 2020b). Smoking reduces the concentration of OPG and, therefore, can be linked to increased osteoclast differentiation and bone resorption (Lappin et al. 2007). This may be similar for vape users given the findings included in this review, whereby RANKL increased, and OPG decreased over time. However, more studies evaluating how vaping may affect the RANK, RANKL, and OPG balance require further investigation.

In this study, vape users did not exhibit as severe periodontal parameters or immunological biomarkers as cigarette smokers; however, they still presented with generalized poorer outcomes in comparison to non‐smokers. It is important to note that cigarette smokers had a longer duration of smoking compared to the duration of vaping among the study participants. The more prolonged exposure might have contributed to the more severe periodontal and immunological biomarkers observed in cigarette smokers. The duration and frequency of use may have influenced treatment response, potentially making vape users appear more responsive than cigarette smokers.

### 4.1 Limitations of Studies Included

When diagnosing periodontal disease and peri‐implant diseases, it is crucial to adhere to clear guidelines to ensure accurate disease identification, communication of disease stages, and appropriate treatment planning, as outlined in the latest classification scheme for periodontal and peri‐implant diseases and conditions (Caton et al. 2018; Tonetti et al. 2018). While some studies employed the latest classification scheme (Al Deeb et al. 2020a; AlJasser et al. 2021; Shah et al. 2023), others did not (Al Deeb et al. 2020b; Al‐Hamoudi et al. 2020; ALharthi et al. 2019; Alhumaidan et al. 2022). The use of differing diagnostic criteria, including the absence of the 2017 World Workshop classification in some studies, likely introduced variability in baseline disease severity (Caton et al. 2018; Romandini et al. 2024). As treatment response in periodontal and peri‑implant therapy varies significantly with disease severity, this heterogeneity may have influenced outcomes and biased inter‑study comparisons. Consequently, differences in staging and grading limit comparability and reduce the generalizability of the conclusions, underscoring the need for future studies to adopt standardized classification systems. In addition, some studies lacked a precise definition of smoking (Alhumaidan et al. 2022) while others did not specify whether vape users had previously used tobacco products, or if vapes included nicotine (Al Deeb et al. 2020a, 2020b; Al‐Hamoudi et al. 2020; ALharthi et al. 2019; Alhumaidan et al. 2022; AlJasser et al. 2021). Due to the nature of vaping and the lack of regulation surrounding e‐cigarette products, the presence or concentration of nicotine in vapes was not discussed in several studies. Furthermore, the absence of standardized guidelines for measuring vaping exposure resulted in inconsistent reporting, with studies using varying metrics such as number of puffs or frequency of daily use (Al Deeb et al. 2020a, 2020b; Al‐Hamoudi et al. 2020; ALharthi et al. 2019; Alhumaidan et al. 2022; AlJasser et al. 2021).

In addition, all smoking data were self‐reported by patients, which may not accurately reflect actual cigarette or vaping consumption and may lead to underreporting. One study included e‐cigarette users with a history of conventional smoking without clearly defining the duration between smoking cessation and initiation of e‐cigarette use (Shah et al. 2023). Others did not specify whether vape users had previously used tobacco products (Al Deeb et al. 2020a, 2020b; Al‐Hamoudi et al. 2020; ALharthi et al. 2019; Alhumaidan et al. 2022; AlJasser et al. 2021). Although all studies stated that the non‐smoker group had never consumed tobacco products, most did not clarify whether non‐smokers had previously used vape products (Al Deeb et al. 2020a, 2020b; Al‐Hamoudi et al. 2020; ALharthi et al. 2019; AlJasser et al. 2021). As a result, the validity of these studies is limited by the varying criteria used to define smokers, vape users, and non‐smokers, thereby reducing comparability between studies and limiting the generalizability of the findings.

A further limitation of this systematic review is the limited number of studies meeting the inclusion criteria that specifically investigated the effects of vaping on surgical periodontal and peri‐implant treatments, restricting the ability to draw definitive conclusions regarding these interventions. Moreover, the included studies did not uniformly control for confounding factors such as oral hygiene practices, socioeconomic status, cultural background, and comorbidities, which could have impacted the results. The absence of randomized controlled trials (RCTs) evaluating the impact of vaping on periodontal and peri‐implant treatment outcomes also hinders the ability to conduct a meta‐analysis, which would provide more robust support for the observed trends. One significant limitation of the study design is the insufficient number of RCTs, with only one RCT included. To comprehensively address the PICO question, a series of RCTs would have been necessary; therefore, the findings of this review must be interpreted with caution. The heterogeneity of the included studies, particularly in terms of study design, participant characteristics, and outcome measures, further complicates the generalizability of the findings. Most of the included studies were conducted in a single geographic region (Saudi Arabia) (Al Deeb et al. 2020a, 2020b; Al‐Hamoudi et al. 2020; Alhumaidan et al. 2022; AlJasser et al. 2021), which limits the external validity of the findings. Population‐specific behaviors, cultural practices, and regional patterns of vaping and smoking in this setting may not reflect those of broader or more diverse global populations, making comparisons and generalizations of outcomes more challenging.

In addition, reliance on a limited number of studies with relatively small sample sizes and varying durations of vaping exposure diminishes the strength of the evidence, highlighting the need for further high‐quality research to clarify the long‐term effects of vaping on periodontal and peri‐implant health.

The studies included a disproportionately higher number of male participants, failing to consider potential systemic hormonal changes that females experience, which may place them at higher risk for soft tissue inflammation and crestal bone loss (Al‐Aali et al. 2018b). Furthermore, all studies employed strict inclusion and exclusion criteria, excluding participants with systemic conditions such as diabetes mellitus, immunocompromising conditions, and those on medications such as steroids, bisphosphonates, or non‐steroidal anti‐inflammatory drugs (NSAIDs), in order to limit potential confounding factors. Overall, the certainty of the available evidence should be considered low to very low, primarily due to the limited number of included studies, predominance of observational designs, heterogeneity in vaping exposure definitions and outcome measures, and relatively short follow‐up periods.

## 5 Conclusions

### 5.1 Implications for Research

The current evidence on the long‐term effects of vaping on periodontal and peri‐implant health remains limited and heterogeneous. With only a small number of studies available, most of which are observational, definitive conclusions about the impact of vaping on treatment outcomes cannot yet be drawn. The findings of this review suggest possible associations between vaping and less favorable clinical and immunological responses compared with non‐smokers; however, these patterns should be interpreted cautiously given the variability in study design, exposure definitions, and outcome measures. There is a critical need for well‐designed longitudinal and interventional studies with larger and more diverse populations to clarify the extent to which vaping influences periodontal and peri‐implant disease progression and treatment response. Future research should also investigate the specific constituents of vape aerosols that may affect host immune responses, thereby improving understanding of the mechanisms underlying these observed associations.

### 5.2 Implications for Clinical Practice

Although the current evidence does not allow for definitive causal claims, the available studies indicate that vape users may experience periodontal and peri‐implant treatment outcomes that trend similarly to those observed in cigarette smokers, whereas non‐smokers consistently demonstrate more favorable results. These preliminary associations highlight the importance of clinicians approaching vaping with the same caution applied to conventional smoking. Until stronger evidence becomes available, healthcare professionals should continue to prioritize smoking cessation counseling and clearly communicate that vaping should not be considered a proven safer alternative in the context of periodontal or peri‐implant therapy. In addition, the current evidence does not support vaping as an effective harm reduction strategy within the context of periodontal or peri‐implant therapy. Ongoing research will be essential to determine the true magnitude of risk associated with vaping and to refine clinical recommendations accordingly.

## Author Contributions

**Stacey May Gould:** data curation, formal analysis, investigation, writing – original draft, software. **Catherine Anne Gilchrist:** data curation, investigation, writing – original draft, software. **Hannah Dominique Gaza:** data curation, investigation, writing – original draft, software. **Ibadat Kaur Sidhu:** data curation, investigation. **Kayleigh Jade Hugo:** data curation, investigation, writing‐original draft, software. **Rodrigo Rodrigues Amaral:** formal analysis, investigation, methodology, software, validation, visualization, writing – review and editing. **João Martins de Mello Neto:** formal analysis, investigation, methodology, project administration, resources, supervision, validation, visualization, writing – review and editing.

## Funding

The authors have nothing to report.

## Ethics Statement

This systematic review was conducted using publicly available data from previously published studies. No new data were collected from human participants or animals; therefore, ethical approval and informed consent were not required. The review adhered to established guidelines for systematic reviews, including transparency, accuracy, and integrity in reporting. All included studies were evaluated for ethical compliance as reported by their original authors.

## Conflicts of Interest

The authors declare no conflicts of interest.

## Supporting information


Supporting File

## Data Availability

The data that support the findings of this study are available on request from the corresponding author. The data are not publicly available due to privacy or ethical restrictions.
