# Supplementary material for: Effect of Vaping on Treatment Outcomes in Patients With Gingivitis, Periodontitis, and/or Peri‐Implant Disease: A Systematic Review
Source: Clin Exp Dent Res. 2026 Sep 15;12(5):e70456. doi: 10.1002/cre2.70456 (PMC13577596; doi:10.1002/cre2.70456)
Supplement: Supplementary file 1 [file CRE2-12-e70456-s001.docx]

**Supplementary Tables and Figures.**

***Table S1: Demonstrating the MeSH and Keywords used in retrieving the reports.***

| **Topics** | **Vape** | **MeSH:**  Vaping, Electronic Nicotine Delivery System  **Keywords:**  vaping OR e-cigarette* OR e cigarette*OR "electronic cigarette*" OR "electronic nicotine delivery system" OR ENDS or e-cigar* OR "vape pen*" OR "personal vaporizer*" OR mods OR vape* OR e-cig* OR "sub-ohms" OR e-hookah* OR "tank system*" OR "nicotine vap*" OR "THC vap*" |
| --- | --- | --- |
|  | **Periodontitis** | **MeSH:**  Periodontitis; Chronic periodontitis; Periodontal pocket; Aggressive periodontitis; Periapical periodontitis  **Keywords:**  "chronic periodont*" OR "adult periodont*" OR "periodont* pocket*" OR "periodont* disease" OR "alveolar bone loss" OR "clinical attachment loss" OR CAL OR "periodontal attachment loss" OR PAL OR "periodontal ind*" OR periodont* OR "periapical periodont*" OR "aggressive periodont*" OR periodontitis OR “periodontal pocket” OR “periodontal disease” |
|  | **Peri-implant disease** | **MeSH:**  Peri-implantitis; Dental implant  **Keyword:**  Peri-implantitis OR periimplantitis OR “peri-implant disease” OR “periimplant disease” OR “peri-implant inflam*” OR “periimplant inflam*” OR “peri-implant mucositis” OR “periimplant mucositis” OR “dental implant” OR inflam* |
|  | **Gingivitis** | **MeSH:**  Gingivitis  **Keyword:**  Gingivitis OR “gingival ind*” OR “gingival pocket” OR “gingival inflam*” OR “gingival inflammation” |
|  | **Treatment** | **MeSH:**  Ultrasonic; Periodontal debridement; Root planing; Subgingival curettage; Dental scaling; Dental prophylaxis; Subgingival scaling; Lithotherapy; Piezosurgery; Guided tissue regeneration; Photochemotherapy; Surgical flap; Gingivoplasty; Laser therapy  **Keyword:**  "Hand sonic" OR "manual sonic" OR ultrasonic* OR "ultrasonic therap*" OR  “sonic oscillat*” OR non-surg* OR  debrid* OR scaling OR “dental scal*” OR SRP OR "scaling and root planing" OR "scal* and polish" OR "root planing" OR prophylaxis OR " operative surgical procedure" OR treatment OR therapy OR curett* OR litho* OR GTR or "guided tissue regenerat*" OR gingivoplast* OR graft OR "bone regenrat*" OR piezo* OR osteoplast* OR "osseous surg*" OR "laser surg*" OR "laser therap*" OR "laser ablation*" OR laser OR photochemotherap* OR “surgical flap*” OR “Periodontal debrid*” OR “subgingival curet*” |

***Table S2: Example of database search strategy.***

| **Database** | **Search strategy** |
| --- | --- |
| Medline [PubMed] | ((((Gingivitis OR "gingival ind*" OR "gingival pocket" OR "gingival inflam*" OR "gingival inflammation") OR ("chronic periodont*" OR "adult periodont*" OR "periodont* pocket*" OR "periodont* disease" OR "alveolar bone loss" OR "clinical attachment loss" OR CAL OR "periodontal attachment loss" OR PAL OR "periodontal ind*" OR periodont* OR "periapical periodont*" OR "aggressive periodont*" OR periodontitis OR "periodontal pocket" OR "periodontal disease")) OR (Peri-implantitis OR "peri implantitis" OR "peri-implant disease" OR "periimplant disease" OR "peri-implant inflam*" OR "periimplant inflam*" OR "peri-implant mucositis" OR "periimplant mucositis" OR "dental implant" OR inflam*)) AND ("vaping" OR e-cigarette* OR e cigarette*OR "electronic cigarette*" OR "electronic nicotine delivery system" OR ENDS or e-cigar* OR "vape pen*" OR "personal vaporizer*" OR mods OR vape* OR e-cig* OR e-hookah* OR "tank system*" OR "nicotine vap*" OR "THC vap*")) AND (("Hand sonic" or ultrasonic* or "ultrasonic therap*" or "sonic oscillat*" or non-surg* or debrid* or scaling or "dental scal*" or SRP or "scaling and root planing" or "scal* and polish" or "root planing" or prophylaxis or " operative surgical procedure" or treatment or therapy or curett* or litho* or GTR or "guided tissue regenerat*" or gingivoplast* or graft or "bone regenrat*" or piezo* or osteoplast* or "osseous surg*" or "laser surg*" or "laser therap*" or "laser ablation*" or laser or photochemotherap* or "surgical flap*" or "Periodontal debrid*" or "subgingival curet*"))  Filters: English, Humans |
